# Effects of propofol and sevoflurane on metabolic changes and oxidative stress markers in laparoscopic appendectomy in children: Clinical control trial

**DOI:** 10.5937/jomb0-56035

**Published:** 2025-08-21

**Authors:** Galambos Izabella Fabri, Biljana Drašković, Marina Pandurov, Goran Rakić, Nikola Bošković, Sanja Vicković, Dragana Pap, Anna Uram-Benka

**Affiliations:** 1 University of Novi Sad, Faculty of Medicine, Novi Sad; 2 Institute for Children and Youth Health Care of Vojvodina, Novi Sad; 3 University of Novi Sad, Faculty of Medicine, Novi Sad, Serbia; 4 University Clinical Center of Vojvodina, Novi Sad; 5 Students Health Protection Institute, Faculty of Pharmacy, Novi Sad

**Keywords:** appendectomy, laparoscopy, pediatric anaesthesia, apendektomija, laparoskopska hirurgija, pedijatrijska anestezija

## Abstract

**Background:**

Laparoscopic appendectomy offers several advantages over open surgery, including shorter recovery time and less postoperative pain. However, it also poses some risks due to the creation of pneumoperitoneum. Understanding and mitigating this risk through the use of appropriate anaesthetic agents, antioxidant therapy, and optimised surgical techniques can improve patient outcomes. This study aimed to determine which of the two anaesthetics, propofol or sevoflurane, provides better suppression of oxidative, inflammatory and metabolic responses in children undergoing laparoscopic or conventional appendectomy.

**Methods:**

This randomised, clinical control trial enrolled 120 children aged 7 to 17 years with a diagnosis of acute appendicitis. Key parameters, including blood glucose, white blood cells, IL-6, oxidative stress markers, lactate, pH, and mean arterial pressure, were analysed.

**Results:**

A statistically significant change in lactate and mean arterial pressure values was observed over time in all four study groups. However, neither the type of surgical procedure nor the type of anaesthesia significantly influenced glucose, white blood cells or thiobarbituric acid levels. The postoperative values of IL-6 were lower in patients treated with balanced anaesthesia.

**Conclusions:**

Our study demonstrated that the type of anaesthesia affects hemodynamic changes, which are closely related to the occurrence of reperfusion injury. Although markers of tissue hypoxia and acidosis were elevated during laparoscopic surgery, their values quickly normalised in the immediate postoperative period.

## Abstract (sr)

**Uvod:**

Laparoskopska apendektomija nudi nekoliko prednosti u odnosu na otvorenu hirurgiju, uključujući kraće vreme oporavka i manji postoperativni bol. Međutim, ona takođe nosi određeni rizik zbog stvaranja pneumoperitoneuma. Ublažavanje ovog rizika upotrebom odgovarajućih anestetičkih agenasa i optimizovanih hirurških tehnika može poboljšati ishode lečenja pacijenata. Cilj ovog istraživanja bio je da se utvrdi koji od dva anestetika, propofol ili sevofluran, bolje potiskuje oksidativne, inflamatorne i metaboličke odgovore kod dece koja se podvrgavaju laparoskopskoj ili konvencionalnoj apendektomiji.

**Metode:**

Ova randomizovana klinička studija je obuhvatila 120 dece uzrasta od 7 do 17 godina sa dijagnozom akutnog apendicitisa. Analizirane su vrednosti glukoze u krvi, bele krvne ćelije, IL-6, markeri oksidativnog stresa, laktat, pH i srednji arterijski pritisak.

**Rezultati:**

Statistički značajna promena u vrednostima laktata i srednjeg arterijskog pritiska zabeležena je tokom vremena u sve četiri ispitivane grupe bolesnika. Međutim, niti tip hirurškog postupka niti tip anestezije nisu značajno uticali na nivo glukoze, belih krvnih zrnaca ili nivoe tiobarbiturne kiseline. Postoperativne vrednosti IL-6 bile su niže kod pacijenata koji su lečeni balansiranom anestezijom.

**Zaključak:**

Naše istraživanje je pokazalo da tip anestezije utiče na hemodinamske promene, koje su usko povezane sa pojavom reperfuzionih povreda. Iako su markeri tkivne hipoksije i acidoze bili povišeni tokom laparoskopske hirurgije, njihove vrednosti su se brzo normalizovale u neposrednom postoperativnom periodu.

## Introduction

The appendix is described as having an early immunological role, particularly significant in children and adolescents [1]. Appendicitis most frequently affects young people and children aged 10 to 19 years, making appendectomy the most common emergency surgical procedure in this age group [2].

Laparoscopic appendectomy is associated with pathophysiological changes that arise from the creation of pneumoperitoneum with gas (usually carbon dioxide, CO_2_) to create a working space for surgery [3], which results from increased intra-abdominal pressure, systemic absorption of the insufflated gas, and the patient's positioning [4]. This leads to reduced mesenteric blood flow and the development of tissue acidosis [5]. Upon desufflation, blood flow is restored, which can lead to a burst of free radicals [6]. This phenomenon is known as ischemia-reperfusion injury, where the sudden return of oxygen to the tissues triggers the formation of reactive oxygen species (ROS) [7]. The surgical trauma itself can activate immune responses, further contributing to the production of free radicals. Volatile anaesthetics are known as cardioprotective and bronchodilator agents, with sevoflurane particularly noted for its ability to attenuate lipid peroxidation [8] [9] [10]. The use of total intravenous anaesthesia (TIVA) with propofol, a lipid-soluble emulsion, during laparoscopy has proven to be a technique associated with good hemodynamic stability in patients [11] and effective in preventing damage caused by reperfusion and ischemia [12]. The reduction of the duration of pneumoperitoneum, by using the lowest effective insufflation pressures, and optimising patient positioning, can help reduce the extent of ischemia-reperfusion injury [13].

Tissue trauma resulting from surgical intervention triggers a complex inflammatory response primarily driven by growth factors and cytokines. By measuring cytokine concentrations in the serum, it is possible to assess the extent of tissue damage from surgical trauma adequately. IL-6 is a cytokine released by macrophages and T cells in response to tissue damage or infection and is specific for assessing the extent of tissue trauma [14] [15]. During laparoscopy, a strong immune response is triggered, the intensity of which is significantly enhanced by the creation of pneumoperitoneum and abdominal distension [16].

Understanding and mitigating these effects through the use of appropriate anaesthetic agents, antioxidant therapy, and optimised surgical techniques can help improve patient outcomes. This study aimed to determine which of the two applied anaesthetics (propofol or sevoflurane) has a better suppression of oxidative and metabolic response in children during laparoscopic or conventional appendectomy. Analysing haemodynamic changes and the impact of two surgical techniques were evaluated as a secondary outcome.

## Materials and methods

This study is a randomised, clinical control trial in children undergoing general anaesthesia for appendectomy. The study was conducted at the Pediatric Surgery Clinic of the Institute for Healthcare of Children and Youth of Vojvodina in Novi Sad, Serbia, between April 2021 and March 2022. The study was performed in accordance with the criteria set by the Declaration of Helsinki and received approval from the Ethic Committee of the Institute for Healthcare of Children and Youth of Vojvodina (442/2021). All patients included in the study had a signed parental or legal guardian's consent for participation in the study before the planned surgical intervention. The study included 120 children scheduled for appendectomy.

The patients were assigned into four groups: laparoscopic appendectomy with propofol (LP group; n = 40), laparoscopic appendectomy with sevoflurane (LS group; n = 40), open appendectomy with propofol (OP group; n = 20) and open appendectomy with sevoflurane (OS group; n = 20). The randomisation process was as follows: the head of the statistics department was responsible for concealing the allocation sequence by using sealed envelopes with sequence and arrangement, and the patients did not know to which group they belonged. The box containing 120 envelopes with allocation instructions (LF) LS, OP and OS group) was used to allocate patients randomly. Inclusion criteria were: children aged 7 to 17 years, with acute non-perforated appendicitis, with a normal body weight (relative to body mass index), without significant comorbidities or on regular medication therapy, ASA I and II. Exclusion criteria included acute and chronic comorbidity, receiving regular medication therapy, over- or underweight children, or perforated gangrenous appendicitis.

For our research, we have initially identified 127 children scheduled for appendectomy. However, seven parents declined to participate, resulting in a total of 120 children who provided informed consent, which were included in the study. All children were examined by an anesthesiologist preoperatively. They received an infusion of crystalloid solution, and the usual fasting regimen was followed [17] prior to the intervention. They were premedicated with intravenous midazolam 0.1 mg/kg. Induction of anaesthesia was intravenous with propofol 2 mg/kg, fentanyl 1 pg/kg, and rocuronium bromide 0.6-1 mg/kg [18]. The airway was secured with an endotracheal tube with a cuff. Mechanical ventilation was pressure- or volume-controlled, with a mixture of air: O_2_ = 50:50, a breathing volume of 6-8 mL/kg and a frequency appropriate for the child's age, maintaining exhaled CO_2_ (EtCO_2_) within reference ranges.

Anesthesia maintenance in Groups LP and OP was carried out with propofol 8-10 mg/kg/h. Depth of anaesthesia was maintained in relation to the values of the Bispectral index (40-50) (Medtronic, Covidien LLC, Mansfield, MA, USA). In subjects of LS and OS groups, anaesthesia was maintained with the inhalation of sevoflurane anaesthetic in concentrations that were needed to maintain the optimal minimal alveolar concentration (MAC) values. In all of the groups, in case of increased pulse or blood pressure for 20% from basal values, bolus doses of fentanyl were given 1 pg/kg. Rocuronium bromide was repeated in case of need in relation to the neuromuscular Train-of-four monitoring as a single dose of 0.2 mg/kg. In cases where it was necessary, on emergence, a reversion of neuromuscular block was administered with neostigmine. Ketorolac was used as an initial postoperative analgesic at a dose of 0.5 mg/kg. All patients received antimicrobial therapy in the postoperative period.

Sampling of blood drawn from the patients was performed three times: a.) 10 minutes after the induction of general anaesthesia; b.) at the moment of releasing air from the abdominal cavity in groups LP and LS, that is, at the moment of removing the appendix from the abdominal cavity in groups OP and OS; and c.) 12 hours after the end of the operation in all studied groups.

EDTA-containing vacuated tubes (Becton Dickinson, San Antonio, TX, USA) were used for blood collection and to prevent blood clotting during the process. Right after, the blood samples were centrifugated (Hettich, ROTOFIX 52A, Burladingen, Germany) at 2400 rotations per minute for 5 min for biochemical analyses.

The leukocyte count was determined by the Advia 212 0 i haematology analyser (Siemens, Germany), which uses a standard and well-established technique of flow cytometry after the specific staining.

Blood for analysis (0.5 mL per sample) was taken from the intravenous cannula, and it was placed in EDTA - containing vacuated tubes (Becton Dickinson, San Antonio, TX, USA). Right after, the blood samples were centrifugated (Hettich, ROTOFIX 52A, Burladingen, Germany) at 2400 rotations per minute for 5 min for biochemical analyses. The obtained plasma was stored at -20°C for a period shorter than 5 months. The analysed parameters related to oxidative stress were the index of lipid peroxidation, determined indirectly through the product of the reaction of lipid peroxidation with thiobarbituric acid (TBARS - Tiobarbituric Acid Reactive Substances). TBARS levels were analysed by Agilent 8455 ultraviolet-visible spectrophotometry system (Agilent Technologies, USA) in the Department of Pharmacy, Faculty of Medicine, University of Novi Sad, Serbia. The results were expressed in nmol/mg protein. The level of the inflammatory response to stress was monitored by analysing IL-6 and the leukocyte count (determined by the Advia 2120i haematology analyser (Siemens, Germany), which uses a standard and well-established technique of flow cytometry after the specific staining). IL-6 (pg/mL) was determined using the ELISA method (manufacturer Bioscience® Platinum ELISA Tests, Human IL-6 High Sensitivity, BMS215HS), using previously frozen serum stored at -20°C. Blood gas analysis with glucose values and lactate and blood count were done immediately from whole blood samples with the anticoagulant EDTA, namely glucose on a Biosen device, and blood count on a haematology counter with 18 parameters Micros ES 60, France. Non-invasively measured arterial blood pressure (NBP) was monitored on a General Electrics Dash 2000 clinical anaesthesia monitor.

The IBM SPSS 27.0 Statistics software package was used for statistical data processing.

Study outcomes were as follows: to evaluate the effect of anaesthetics on oxidative stress, we measured TBARS, and to evaluate the effect on metabolic stress, we measured glucose, lactate, leucocytes and IL-6. Secondary outcomes were to assess the effects of the type of surgery on the abovementioned parameters.

Descriptive statistics was used to determine the mean value and standard deviation, and Student's t-test was used depending on the variables. The non-parametric Mann-Whitney U test was used to analyse the influence of anaesthetics and the type of surgery on the values of IL6. One factor analysis of variance (ANOVA) of different groups with subsequent tests was used to analyse differences in age and weight of patients. The Kruskal-Wallis test for categorical variables was used to analyse the duration of procedures.

One-factor analysis of variance with repeated measurements was used to test the changes in parameters in the observed time interval. Tukey, LSD Post Hoc test of pairs of groups was used to determine between which groups these differences were statistically significant. One factor analysis of covariance (ANCOVA) was used to test the parameters that varied at the initial measurement. Friedman's test for categorical variables was used to determine the differences in interval variables during the examined time. Throughout these analyses, significance levels were established at p<0.05, ensuring an evaluation of statistical significance.

## Results

General information about the pediatric patients in the four studied groups, along with their comparative values and statistical significance, are listed below in Table 1 for comparative evaluation. The difference in surgery duration and the mean times required for the surgical removal of the appendix were similar regardless of the surgical method used (Table 2).

**Table 1:** General data about the patients listed in groups. Values are mean ± standard deviation and numbers (%)<br>*ANOVA, **χ^2^

Parameter	LP	LS	OP	OS	p-Value
Age (years)	11.98 ±3.16	10.96±3.09	12.60±2.80	12.40±3.52	0.179*
Body weight (kg)	47.62±17.01	41.43±11.88	45.65±16.61	49.42±19.54	0.214*
Male gender	20 (50%)	20 (50%)	15 (75%)	13(65%)	0.190**
Female gender	20 (50%)	20 (50%)	5 (25%)	7 (35%)	

**Table 2:** Data about the parameters of anesthesia as divided in groups. Values are mean ± standard deviation<br>* Kruskal Wallis test

Parameter	LP	LS	OP	OS	p-Value
Duration of intervention<br>(minutes)	74.75±20.53	74.75±15.97	69.25±16.80	65.00±17.16	0.116*
Appendix removal time<br>(minutes)	49.88±13.84	49.95±12.11	49.95±13.22	48.20±13.87	0.941*

Analysing the values at the initial measurement (ANOVA), MAP values did not differ significantly between the groups (F=1,617; p=0,189), but at the time of appendix removal, it was found that MAP values were significantly lower during laparoscopy in the LS group compared to the LP group (F = 8,05; p=0,000). The changes in MAP values are listed in Table 3.

**Table 3:** Change of MAP in the observed period. Values are mean ± standard deviation

	LP			LS			OP			OS		
	a	b		a	b	c	a	b	c	a	b	c
MAP<br>(mmHg)<br>X	85.13	86.75	77.15	79.28	77.00	74.53	81.20	82.35	79.60	81.60	80.50	79.55
SD	11.31	9.34	7.16	11.16	7.83	8.11	12.45	11.39	9.85	14.54	7.49	10.12

Observing the indicators of lipid peroxidation during appendectomy, we found no statistically significant difference between the examined groups at any moment of the measurement (Table 4).

**Table 4:** Change of parameters during the three observed periods. *One-factor analysis of repeated measurements with Tukey, LSD Post Hoc test of pairs of groups; **Friedman's test

	LP			LS			OP			OS		
	a	b	c	a	b	c	a	b	c	a	b	c
Lactate (mmol/L)	2.27	2.42	1.54	1.96	2.57	1.62	1.94	2.22	1.46	1.75	2.23	1.41
P*(a-b)	0.760			0.000			0.503			0.004		
pH	7.47	7.45	7.42	7.49	7.45	7.43	7.46	7.48	7.42	7.5	7.5	7.43
p*(a-b)	0.120			0.000			0.077			1.000		
Glucose (mmol/L)	5.39	6.05	5.12	5.34	5.51	5.04	5.57	5.98	4.69	5.51	5.91	4.97
p*(a-b)	0.000			1.000			0.003			0.001		
TBARS (nmol/mg)	7.69	7.62	8.84		8.34	8.36	7.92	6.87	8.18	9.44	12.38	10.82
p**	0.422			0.593			0.232			0.779		
WBC (10^9^/L)	9.50	10.55	11.06	8.65	9.14	9.88	14.45	12.93	10.83	15.04	12.72	11.75
p*(a-b)	0.064			0.989			0.732			0.034		

During the analysis of the change in lactate values over time, it was observed that the lactate values in all four observed groups changed. In all four groups, lactates increased during surgery. However, this increase was statistically significant only in the group of patients treated with sevoflurane (LS and OS) (Table 4).

We found that the pH values changed due to the type of surgery (pH dropped during laparoscopy) and that anaesthetics had no effect on the pH values at any moment (Table 4).

By observing the change in glucose values over time, in the LF) OP and OS groups, the glucose values in the intraoperative period (the period between the initial measurement and the moment of appendectomy) increased statistically significantly, only to drop statistically significantly in the postoperative period. During laparoscopy with sevoflurane, glucose did not increase significantly during the operation (Table 4).

Initial values of WBCs were different in different groups of patients. Results of ANCOVA showed that after the statistical removal of the influence of initial values of WBCs at the first measurement (WBC a), there was no difference in the values at the moment of appendix removal (WBC b). Further analysis showed that WBCs did not change significantly during the whole observed period in any of the groups (Table 4).

IL6 values did not change during the intraoperative period, regardless of the type of surgery or anaesthesia used. The Kruskal-Wallis test showed that at 12 hours after the intervention, a highly statistically significant difference in IL-6 concentrations was found between the groups. Subsequent analyses using the Mann-Whitney U test revealed statistically significantly lower IL-6 concentrations 12 hours after the intervention in patients who received sevoflurane compared to those who received propofol, both among patients undergoing laparoscopic surgery and those undergoing open surgery (Table 5).

**Table 5:** Variations of IL-6 during the observed period. *Man-Whitney U test

	LP-LS<br>P*	OP-OS<br>P*	LP-OP<br>P*	LS-OS<br>P*
IL-6 a	0.240	0.337	0.190	0.058
IL-6 b	0.40	0.185	0.082	0.204
IL-6 c	0.001	0.000	0.649	0.082

## Discussion

The patients observed in the study were homogeneous. The duration of laparoscopy was slightly longer than open surgery, but appendix removal time did not differ between groups. During laparoscopy, sevoflurane caused a more significant reduction in blood pressure compared to propofol, which was associated with a higher increase in intraoperative lactate levels, although without significant changes in acid-base balance. Intraoperatively, sevoflurane provided better stress control, as it more effectively suppressed glucose levels than propofol. Neither anaesthetic influenced oxidative stress, but patients treated with sevoflurane exhibited lower postoperative inflammation, as indicated by reduced IL-6 levels, regardless of the type of surgery.

In the research, the dose of propofol used for induction of anaesthesia was usually for pediatric patients [19]. Laparoscopic operations lasted slightly longer compared to classic appendectomies, which is similar to the results of other authors [20].

Standard monitoring included non-invasive measurement of arterial pressure [21]. In the four groups of patients observed, it was determined that children whose anaesthesia during laparoscopy was maintained with propofol had higher MAP values than those who underwent sevoflurane anaesthesia intraoperatively. This had no repercussions in the postoperative period. The results indicated that MAP values were influenced not by the type of surgery but from the kind of anaesthetic used.

The value of glucose increases significantly during laparoscopic surgery as part of the perioperative reaction to surgical stress [22]. An increase in glucose was also recorded in our current study during laparoscopic appendectomy. However, in our research, the difference in glucose increase between open and laparoscopic interventions was not statistically significant [23]. In contrast to the studies mentioned above, the results of our research show that sevoflurane is an anaesthetic that provides good control of blood glucose levels in the perioperative period, as this parameter remained normal during laparoscopic appendectomy [24].

The most common cause of hyperlactatemia, among others, is probably microcirculatory hypoperfusion [25]. The creation of a pneumoperitoneum and the consequent distension of the abdomen result in local hypoperfusion in the splanchnic circulation [20] [26]. In our study, levels of lactates increased during surgery with a decrease in MAP and reached values >2 mmol/L. A change in the value of both parameters was recorded in patients in whom anaesthesia was maintained with sevoflurane during the operation. Similar results were obtained by Carles et al. [27].

A pediatric patient tolerates prolonged action (>120 min) of low-pressure pneumoperitoneum well, but significant changes in hemodynamics, acid-base balance and total blood volume may develop [28]. It was established that during the initial measurement, all patients were in moderate alkalosis. The reason for this result may lie in the fact that all patients were rehydrated with crystalloid solutions before the intervention [29], and some vomited [30]. By observing the kinetics of this parameter, we determined that intraoperative pH dropped statistically significantly during laparoscopy and that this was not the case with classic appendectomies. The type of applied anaesthesia did not affect the change in pH value intraoperatively [31].

Oxidative stress occurs when reactive oxygen species (ROS) exceed the body's antioxidant capacity, primarily generated in the mitochondria [32]. We assumed that all subjects had elevated oxidative stress at the start of the operation due to the nature of appendicitis [33] [34]. At the initial measurement, TBARS values were elevated but did not differ significantly among groups [35]. The pneumoperitoneum increases intra-abdominal pressure, reducing hepatic, splanchnic, and renal circulation, leading to oxidative stress [16] [36]. TBARS values during propofol anaesthesia were lower at the time of appendix removal in both LP and OP groups. With sevoflurane, TBARS remained unchanged during laparoscopic surgery and were higher in open surgery. TBARS kinetics showed no significant increase in oxidative stress markers within 12 hours post-operation [37].

Analysis of leukocyte values in our participants showed no significant difference at the time of appendix removal or 12 hours post-intervention. All participants received antimicrobial therapy, which may have contributed to the uniform decrease in leukocyte values. The type of anaesthesia did not influence the values of WBCs. During laparoscopic surgery, leukocyte count slightly increased but remained within reference values, which is comparable to some authors who reported similar findings [38].

The level of IL-6, a significant inflammatory marker [39], is known to increase 1-3 hours after the start of surgery and can remain elevated for 2-3 days postoperatively. The average duration of appendectomy in our study was 72 minutes, which is enough time for an intraoperative increase in IL-6. Patients who received balanced anaesthesia with sevoflurane had statistically significantly lower IL-6 levels 12 hours after surgery compared to those who received propofol anaesthesia, regardless of the type of surgical procedure. We were unable to confirm these findings in other studies, likely due to the short time interval between the three sampling points observed [40] [41].

The results of our study, in addition to the abovementioned, indicate that the effects of anaesthesia, surgery and the acute disease itself have intertwined effects on the parameters observed. Sevoflurane-based balanced anaesthesia was accompanied by lower blood pressure and somewhat poorer metabolic outcomes intraoperatively, with no repercussions during the postoperative period. The above stated indicates further research, with possibly more reliable results on a larger sample and analysing more outcome parameters such as wound healing, hospitalisation length and recovery time.

## Conclusion

In our study, it was shown that the type of anaesthesia affects changes in hemodynamics, which is directly related to the occurrence of reperfusion damage with little or no effect on the early postoperative period. Markers of tissue hypoxia and acidosis have higher values during laparoscopic surgery; however, their values quickly recede in the immediate postoperative period. Sevoflurane-based anaesthesia may reduce overall postoperative inflammation by efficiently reducing intraoperative stress during laparoscopic appendectomies in children.

## Dodatak

### List of abbreviations

ROS, reactive oxygen species;<br>TIVA, total intravenous anaesthesia;<br>CRP C-reactive protein;<br>IL-6, interleukin 6;<br>TNF-α, tumour necrosis factor alpha;<br>ASA, American Society of Anesthesia physical status classification;<br>MAC, minimal alveolar concentration;<br>TBARS, tiobarbituric acid reactive substances;<br>EDTA, ethylenediamine tetraacetic acid;<br>NBP non-invasively measured arterial blood pressure;<br>SD, standard deviation;<br>MAP mean arterial pressure.

### Conflict of interest statement

All the authors declare that they have no conflict of interest in this work.
